# Optimization of Magnetic Tunnel Junction Structure through Component Analysis and Deposition Parameters Adjustment

**DOI:** 10.3390/ma17112554

**Published:** 2024-05-25

**Authors:** Crina Ghemes, Mihai Tibu, Oana-Georgiana Dragos-Pinzaru, Gabriel Ababei, George Stoian, Nicoleta Lupu, Horia Chiriac

**Affiliations:** National Institute of Research and Development for Technical Physics, 700050 Iasi, Romania

**Keywords:** magnetic tunnel junction, thin films, roughness, tunnel magnetoresistance, sputtering deposition

## Abstract

In this work, we focus on a detailed study of the role of each component layer in the multilayer structure of a magnetic tunnel junction (MTJ) as well as the analysis of the effects that the deposition parameters of the thin films have on the performance of the structure. Various techniques including atomic force microscopy (AFM), scanning electron microscopy (SEM), and transmission electron microscopy (TEM) were used to investigate the effects of deposition parameters on the surface roughness and thickness of individual layers within the MTJ structure. Furthermore, this study investigates the influence of thin films thickness on the magnetoresistive properties of the MTJ structure, focusing on the free ferromagnetic layer and the barrier layer (MgO). Through systematic analysis and optimization of the deposition parameters, this study demonstrates a significant improvement in the tunnel magnetoresistance (TMR) of the MTJ structure of 10% on average, highlighting the importance of precise control over thin films properties for enhancing device performance.

## 1. Introduction

The discovery of the magnetoresistive effect by William Thomson (Lord Kelvin) [1] has aroused the interest of the scientific community, as it can be used in various areas of detection. The magnetoresistive effect involves a change in the electrical resistance of a structure when a magnetic field is applied. This variation is quantified by the magnetoresistance ratio (MR) and can occur in non-magnetic semiconductor–metal hybrid structures [2], metals [3], and magnetic metals [4] or in multilayer systems such as magnetic tunnel junctions (MTJs) and giant-magnetoresistance, colossal-magnetoresistance, and extraordinary-magnetoresistance structures. Among magnetoresistive structures, those based on the tunnel magnetoresistance effect have a higher MR and better spatial resolution [5,6,7], which recommends them for various applications [8,9,10]. One of the most attractive applications of MTJ structures is in the biomedical field [11,12,13], where they are used for various types of sensors.

The typical MTJ structure consists of two ferromagnetic layers separated by a thin insulating layer. Commonly used insulating materials include aluminum oxide (AlOx) and magnesium oxide (MgO). Previous research has shown that MTJ structures with a MgO barrier layer have a higher magnetoresistance [14], which is due to coherent tunneling enabled by the crystalline structure of MgO. The use of MgO as a barrier layer in MTJ-based sensors requires a layer that is as smooth and defect-free as possible, which strongly depends on the quality of the individual component layers on which MgO is deposited.

## 2. Materials and Methods

The magnetic tunnel junction multilayer structures analyzed in this work have a typical composition of Ta (5)/Ru (20)/Ta (5)/CoFe (2.5)/IrMn (20)/CoFe (2.5)/Ru (0.85)/CoFeB (3)/MgO (1.8)/CoFeB (3)/Ta (5), where the numbers in brackets represent the thicknesses in nanometers, and the roles of each layer are presented in Table 1. These structures were deposited onto 18 × 18 mm^2^ thermal oxide silicon (Si/SiO_2_) wafers using an ATC 2200/AJA International deposition system (Scituate, MA, USA) capable of reaching a base pressure of 5 × 10^−8^ Torr. The system enables magnetron sputter discharge with two direct-current (DC) and four radio-frequency (RF) discharge sources, as well as electron-beam evaporation. Except for the barrier layer (MgO), which is deposited by electron-beam evaporation, all other thin films are produced by sputtering. Argon (Ar) was used as the inert gas for plasma generation. During deposition, a magnetic field was applied parallel to the plane of the thin films to define the easy axis of the ferromagnetic layer. The average roughness (R_a_) of each component layer, which indicates the average difference between the peaks and valleys over the measured surface, was determined by scanning the surface at several points with the AFM XE-100 atomic force microscope (Gwanggyo-ro, Suwon, Republic of Korea).

**Figure 1:**
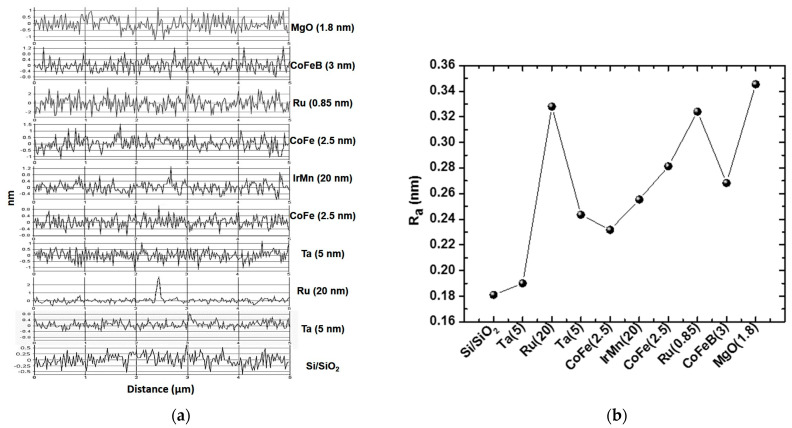
(**a**) Roughness profiles of the thin films surface; (**b**) variation in roughness of the MTJ component layers.

To evaluate the quality of each material deposited under the barrier layer, nine Si/SiO_2_ wafers were placed inside the deposition system. The number of wafers is equal to the number of thin films deposited up to and including the barrier layer. After the deposition of each material within the MTJ structure with the above-specified thicknesses, the average roughness was determined (Figure 1). The average roughness was determined starting from the oxidized silicon substrate layer and then measured after each layer of the structure was deposited. The deposition conditions for the thin films in the MTJ structure, upon which this study was based, included a gas pressure of 5 mTorr, discharge powers of 150 W for DC sputtering and 120 W for RF sputtering, and a target-to-substrate distance of 20 cm.

As shown in Figure 1, with the deposition of each new layer of the MTJ structure, the roughness increases. However, the difference is given by the Ru layer, which exhibits a high average roughness both at a thickness of 20 nm and at 0.85 nm. In Figure 1a, there is a peak in the Ru–20 nm scan that could contribute to a higher roughness, as this peak can be associated with a physical surface roughness, but an increase in roughness is also observed after depositing the Ru–0.85 nm layer. This indicates that the overall roughness of the deposited Ru layer is higher than that of the other materials in the MTJ structure. This discrepancy in roughness could be caused by various factors, such as different material properties or deposition processes. We believe that Ru naturally tends to form rougher surfaces than other materials in MTJs. However, Ru also offers advantages like higher electrical conductivity and greater thermal stability compared to tantalum, making it the most suitable for such applications.

The roughness of the Ru layer, as well as the other layers, can be influenced by the deposition parameters. Therefore, optimizing the deposition process parameters and closely monitoring the deposition conditions are important steps to minimize the roughness and obtain thin films with the desired properties of the magnetoresistive structure.

## 3. Results and Discussion

In order to improve the uniformity of the MgO barrier layer, we analyzed the roughness of the thin films deposited up to the barrier layer as a function of the deposition parameters. Magnetron sputtering techniques (RF and DC) were used to investigate the effects of working gas pressure, discharge power, and distance between target and substrate on the MTJ structure.

### 3.1. Working Gas Pressure

**Figure 2:**
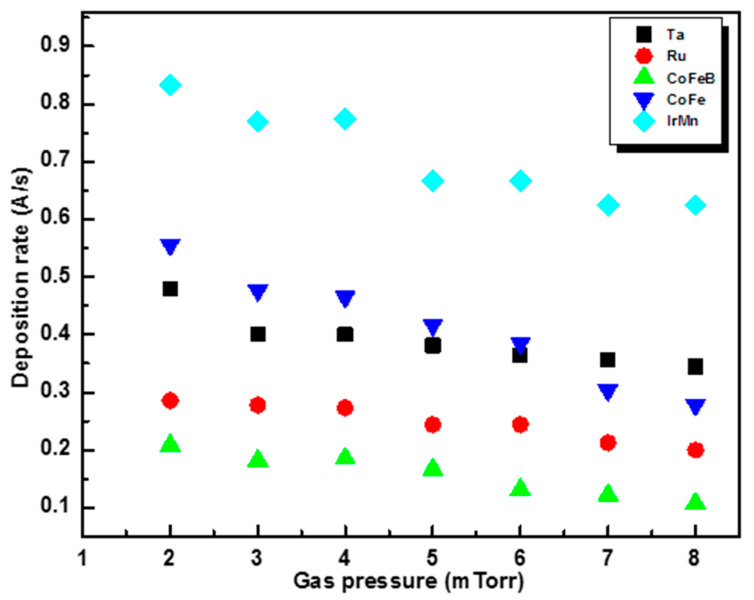
Deposition rate of the component layers as a function of gas pressure.

The deposition rate of the materials varies with the working gas pressure, that is, the flow pressure of the argon gas during deposition, which influences the degree of surface uniformity of the materials. Studies have also shown that due to the lower deposition rate of RF sputtering compared to DC sputtering, the materials deposited by RF sputtering have a lower roughness than the same material deposited by DC sputtering [15,16]. Based on these studies, Ta and Ru were deposited by RF sputtering to ensure a buffer layer that was as uniform as possible. The CoFeB layer was also obtained by RF sputtering, while IrMn and CoFe were obtained by DC sputtering. The deposition rates were evaluated as a function of working gas pressure in the range of 2–8 mTorr for each component layer in the MTJ structure at a discharge power of 120 W (Figure 2). The values of the rates differ from one material to another, which can be explained by the differences in the atomic mass of the materials and the size of the ejected atoms.

**Figure 3:**
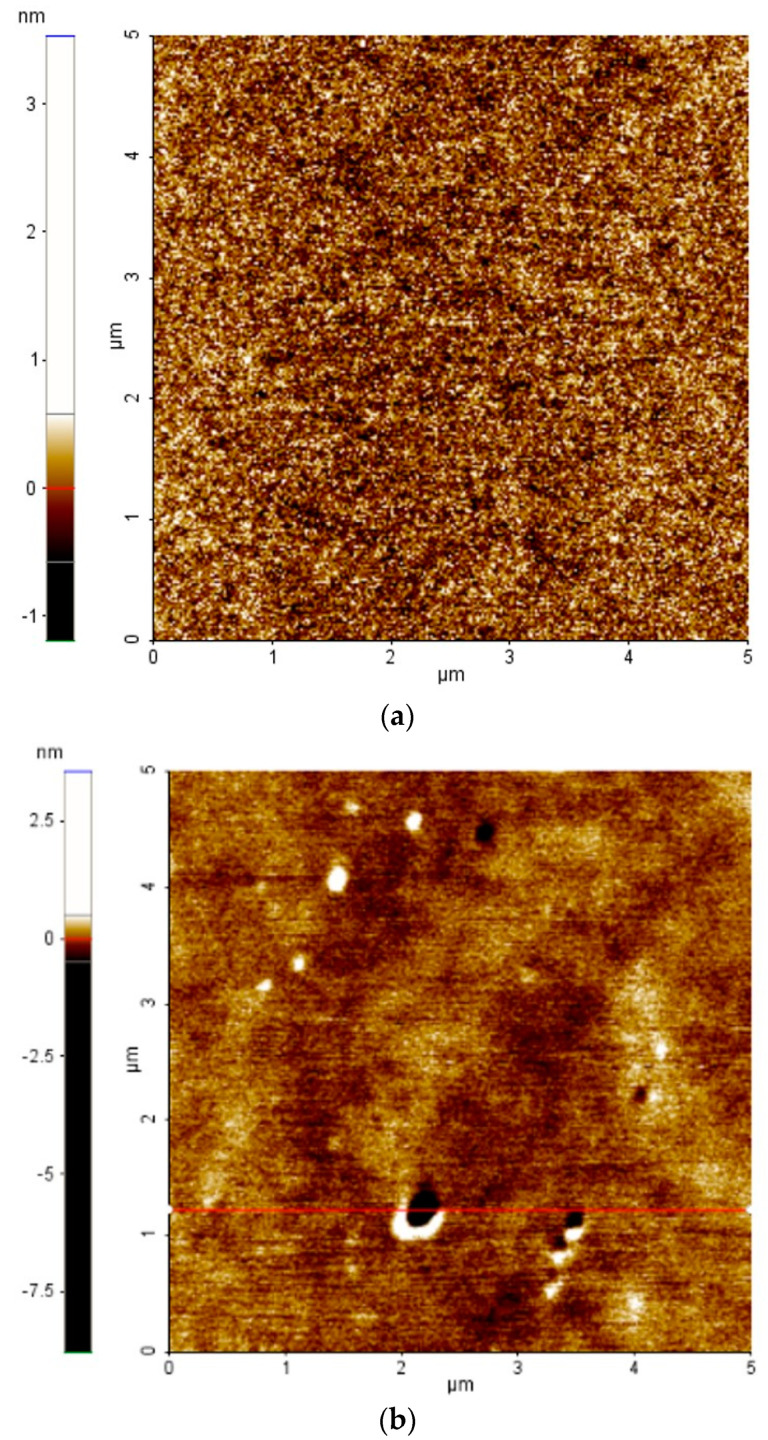
Atomic force microscopy (AFM) topography images of CoFe thin film deposited at a gas pressure of (**a**) 3 mTorr and (**b**) 5 mTorr.

As the results in Figure 2 show, the variation in deposition rate as a function of gas pressure has approximately the same trend for all materials, i.e., the values at 2 mTorr show the highest deposition rate, and at 3 and 4 mTorr, the deposition rates are almost the same, followed by a decrease in deposition rates after 4 mTorr. In both RF and DC deposition, instability of the generated plasma was observed at a working pressure of 2 mTorr, indicating that there are not enough collisions between atoms and electrons to sustain the plasma at this working pressure. On the other hand, as the pressure increases, the deposition rate decreases, which may lead to an increase in the roughness of the thin films, as evidenced by the results of atomic force microscopy (AFM) measurements of the surface of the CoFe film at pressures of 3 and 5 mTorr. After the thin films were scanned at several points, an average roughness (R_a_) of 0.224 nm at 3 mTorr (Figure 3a) and 0.456 nm at 5 mTorr (Figure 3b) was observed.

The measurements show that the working pressure of 3 mTorr is sufficient for activating the plasma and also for maintaining an optimal deposition rate, resulting in a lower surface roughness of the deposited thin film.

### 3.2. Discharge Power

**Figure 4:**
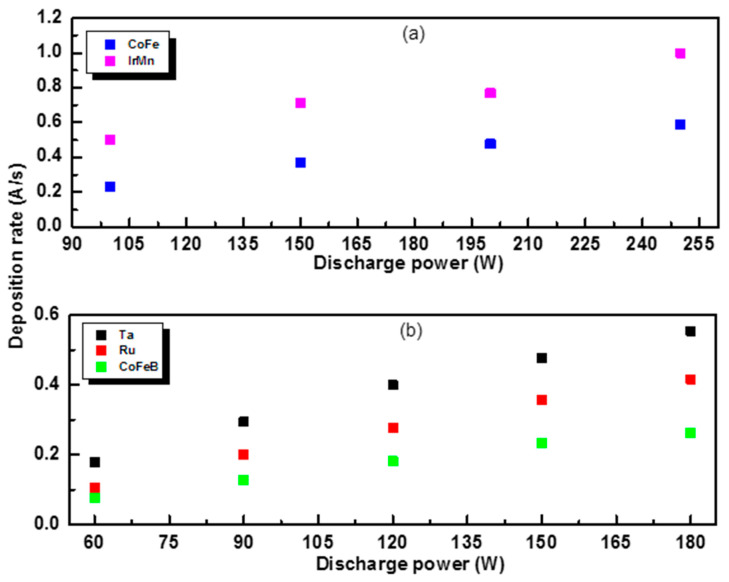
Deposition rate of the component layers as a function of discharge power for DC sputtering (**a**) and RF sputtering (**b**).

The effects of the variation in the discharge power on the deposition rates (Figure 4) and, implicitly, on the surface morphology of the individual layers of the MTJ structure were also analyzed.

The rates increase with the increasing discharge power for each material. According to studies on the influence of discharge power on surface morphology [17,18], at very low powers, the roughness of the deposited thin films increases due to the low kinetic energy of the atoms or ions, which causes them to accumulate in the grains. On the other hand, if the discharge power is very high, the ionized species have high kinetic energy such that the atoms ejected from the target do not have time to rearrange on the substrate until the next atoms arrive, which, in turn, can lead to an increase in the roughness of the deposited film [19].

**Figure 5:**
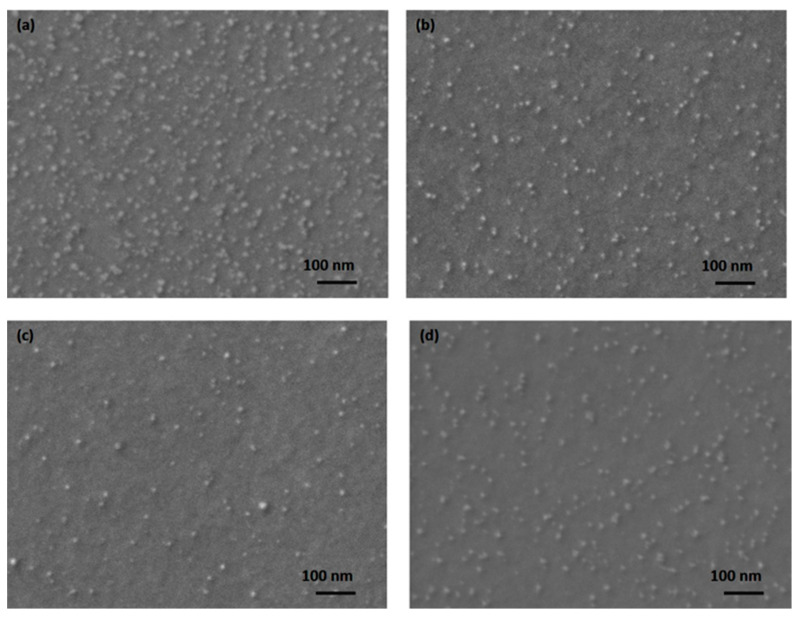
Scanning electron microscopy images of CoFeB surfaces deposited at (**a**) 90 W, (**b**) 120 W, (**c**) 150 W, and (**d**) 180 W.

Considering that the performance of the MTJ structure is strongly influenced by the magnetic properties of the ferromagnetic layers in the structure [20,21], the effect of the discharge power on the morphological structure of the CoFeB thin films was investigated. For this purpose, a series of four samples with CoFeB layers (20 nm) were deposited on Si/SiO_2_ wafers at different discharge powers of 90 W, 120 W, 150 W, and 180 W and then annealed at 320 °C for one hour. Scanning electron microscopy (SEM) analysis using a microscope from Carl Zeiss (Oberkochen, Germany) of the CoFeB surface (Figure 5) shows that the surface of the film deposited at 90 W exhibits a large number of clusters in the form of grains. The number of these grains decreases with increasing discharge power up to 150 W, whereby the surface appears smoother and has fewer grains. At a power higher than 150 W, the number of accumulations increases again, meaning that 150 W is the discharge power at which CoFeB can be deposited at a suitable rate to obtain continuous thin films with minimum roughness.

**Figure 6:**
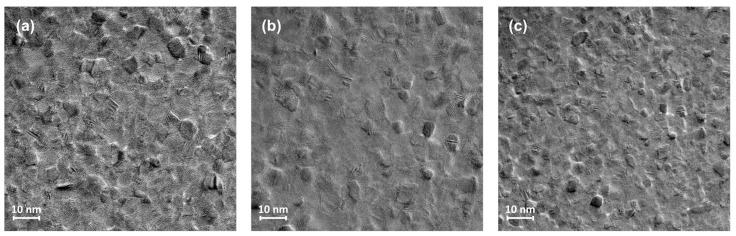
Transmission electron microscopy images of Ru surfaces deposited at discharge powers of (**a**) 120 W, (**b**) 150 W, and (**c**) 180 W.

Because the roughness of the Ru layer increases, as shown by the AFM measurements in Figure 1a, the roughness of the entire MTJ structure also increases. Therefore, we focused on solving this problem. For this purpose, the deposition rate of Ru was varied by changing the discharge power during deposition. More specifically, three samples of Ru thin films with a thickness of 20 nm were sputter-deposited on carbon-supported grids used at discharge powers of 120 W, 150 W, and 180 W (Figure 6) and analyzed by transmission electron microscopy using UHR-TEM model LIBRA^®^ 200 MC from Carl Zeiss GmbH (Jena, Germany).

From the TEM images of the Ru surface, it can be seen that the layer deposited at 150 W (Figure 6b) exhibits a higher degree of uniformity than those deposited at the other two discharge powers (Figure 6a,c), indicating that the roughness of the Ru layer can be reduced by increasing the discharge power from 120 to 150 W.

### 3.3. Target–Substrate Distance

**Figure 7:**
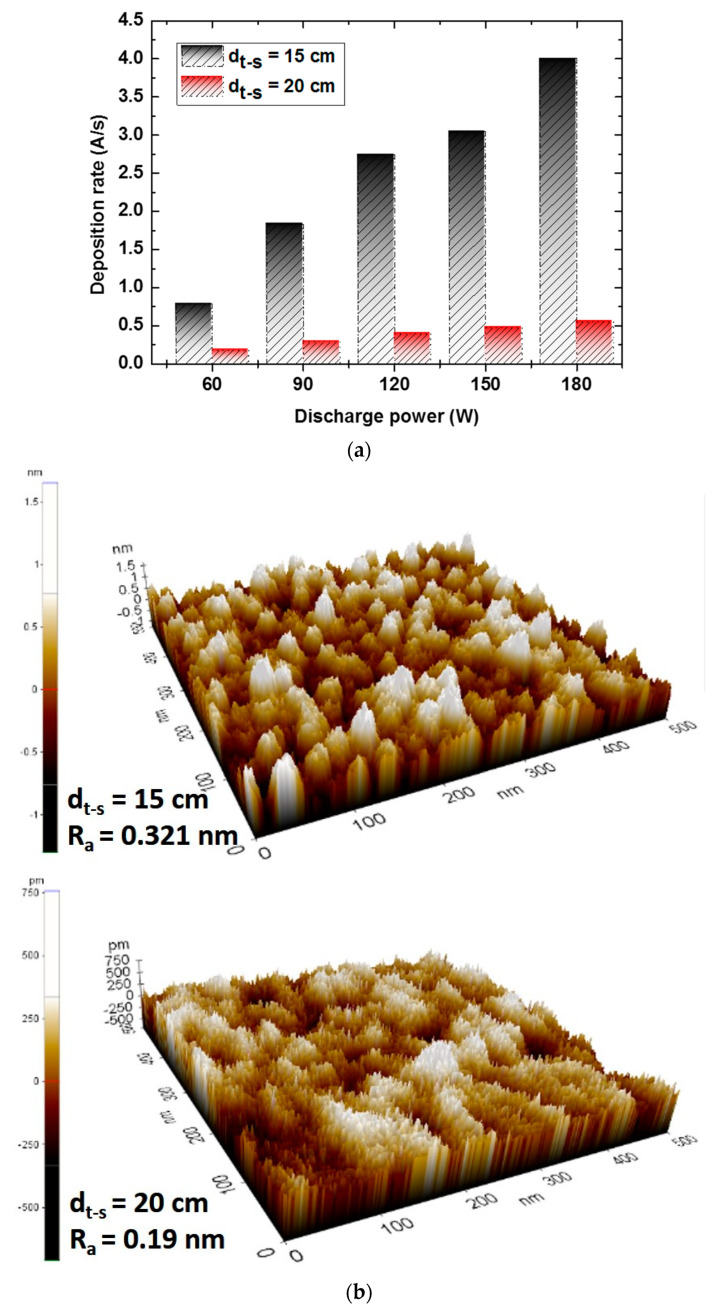
(**a**) Variation in Ta deposition rate at target–substrate distances of 15 and 20 cm; (**b**) topographic AFM images of the surface of the Ta layer (5 nm) deposited at the two distances.

The distance between the target and substrate (d_t-s_) in the sputter deposition process has been widely investigated for several materials [22,23], and it has been demonstrated that this has a great impact on the quality of the deposited thin films. In particular, in this work, we analyzed the surface roughness of the films for d_t-s_ of 15 and 20 cm (Figure 7).

With the decreasing distance between target and substrate, an increase in roughness was observed at a smaller distance, which is due to the increase in the deposition rate of each material. Figure 7a proves that the deposition rates increase at a smaller distance regardless of the discharge power. Also, when evaluating the Ta surface area (Figure 7b), an increase in roughness can be observed at a distance of 15 cm compared to 20 cm. The effect of the distance between the target and the substrate on the roughness was investigated for all materials in the MTJ structure. It was observed that, similar to the Ta layer, the roughness of these materials increased as the distance between the target and the substrate decreased.

**Figure 8:**
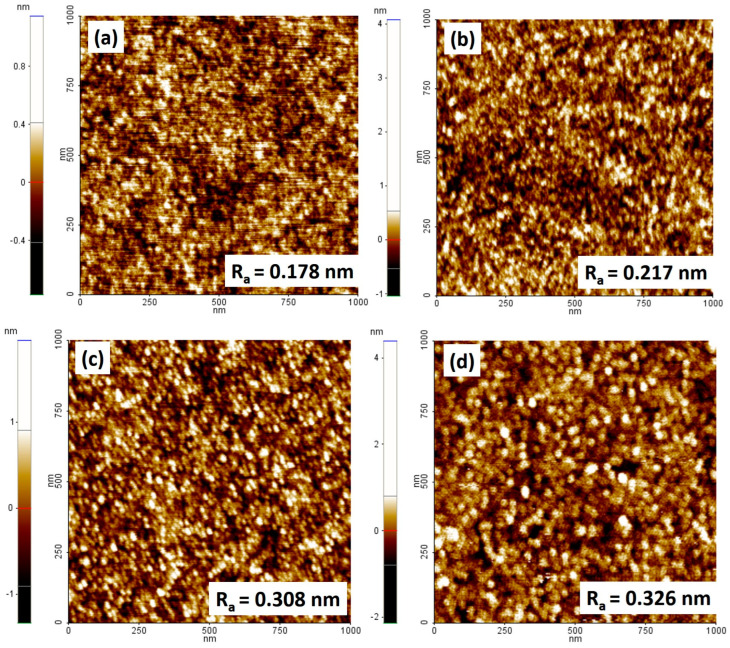
Topographical AFM image of the Ru layer in the synthetic antiferromagnetic structure with a thickness of (**a**) 0.8 nm, (**b**) 0.85 nm, (**c**) 0.9 nm, and (**d**) 1 nm.

To obtain an MTJ structure with high magnetoresistance, the thickness of the Ru layer in the synthetic antiferromagnetic (SAFM) structure must be adjusted to ensure proper antiferromagnetic coupling between the fixed CoFe layer and the CoFeB reference layer through a Ruderman–Kittel–Kasuya–Yosida (RKKY)-type coupling oscillator. Although the strongest coupling can be achieved at a Ru thickness of 0.35 nm, which is at the first RKKY oscillation peak [24], this thickness is difficult to control from a practical point of view. Therefore, the thickness range of 0.8–1 nm, which belongs to the second oscillation peak [25], is preferred. Considering the importance of strong antiferromagnetic coupling, it is essential that the separator layer in the SAFM possesses higher homogeneity and continuity. Therefore, it is necessary to find the thickness of the Ru layer (≤1 nm) at which the roughness is the lowest so that the discontinuities on the surface are minimized. For this purpose, we analyzed the roughness of the Ru surface (Figure 8) in the multilayer structure of the type Ta (5)/Ru (20)/Ta (5)/CoFe (2.5)/IrMn (20)/CoFe (2.5)/Ru (x), where x is the thickness of the Ru layer (x = 0.8, 0.85, 0.9, and 1 nm).

As the AFM images show, a slight increase in roughness can be observed with increasing the thickness of the Ru layer. At a thickness of 0.8 nm, Ru has the lowest average roughness, but the thinner the layer is, the more surface defects (discontinuities) can occur, resulting in a reduction of the antiferromagnetic exchange coupling. Therefore, the Ru layer of 0.85 nm could be an optimal compromise between thickness control and roughness minimization, as this layer thickness is slightly larger compared to 0.8 nm and could provide more precise control over the experimental process. Considering the results from the literature [25,26] where Ru with a thickness of 0.85 nm is used, but also the results from AFM images where the Ru surface is much more uniform at a thickness of 0.85 nm than at 0.9 nm and 1 nm, we assume that 0.85 nm Ru could ensure an optimal coupling between the two ferromagnetic layers for our MTJ structure.

**Figure 9:**
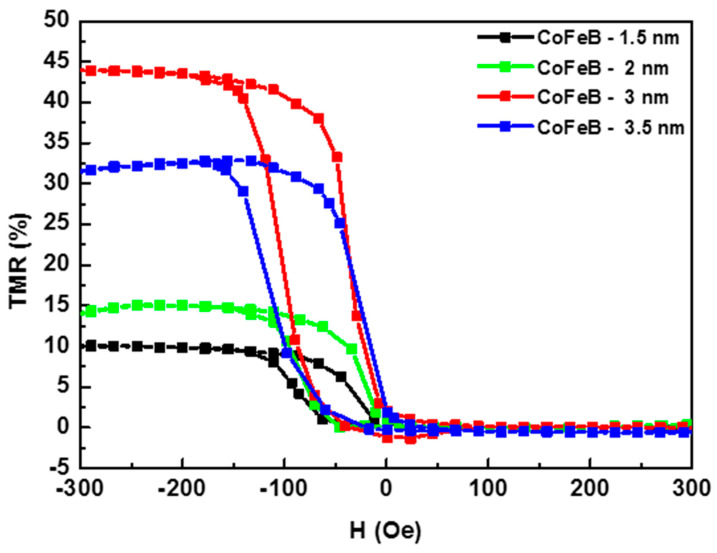
Magnetoresistance curves for MTJ structures as a function of CoFeB layer thicknesses.

Of particular importance in the MTJ structure is the free ferromagnetic CoFeB layer, which has the same thickness as the reference layer. The thickness of the free layer influences the type of response (linear or quadratic) of the sensor to the external magnetic field [27]. The type of response also depends on the shape and dimensions of the sensor, but in this work, we were only interested in the effect of the thickness on the magnetoresistance ratio and did not aim to obtain a linear response. To investigate the influence of the thickness of the free ferromagnetic layer, the electrical resistance of the MTJ structure was recorded as a function of the applied magnetic field. This change is referred to as the tunnel magnetoresistance (TMR) and was measured for a series of samples with a multilayer structure of the type Ta (5 nm)/Ru (20 nm)/Ta (5 nm)/CoFe (2.5 nm)/IrMn (20 nm)/CoFe (2.5 nm)/Ru (0.85 nm)/CoFeB (x nm)/MgO (2 nm)/CoFeB (x nm)/Ta (10 nm), where the thickness of the CoFeB layer was varied (x = 1.5 nm, 2 nm, 3 nm, 3.5 nm). For the TMR measurements, it was necessary to microfabricate the MTJ-based sensors in the CPP configuration (current perpendicular to plane). In the CPP geometry, the electric current flows perpendicular to the plane of the MTJ multilayer structure and enables the efficient detection of changes in resistance with external magnetic fields, making it a crucial element in various magnetic sensing applications [28,29]. After microfabrication of the MTJ sensors, the samples were annealed in a magnetic field for one hour to crystallize the CoFeB layer and fix the magnetization in the SAFM layer. The measurements were carried out in a homogeneous magnetic field with a maximum amplitude of ±300 Oe, which was generated by a Helmholtz coil system. The TMR curves (Figure 9) were obtained by measuring the electrical resistance of the samples as a function of the applied magnetic field.

A clear difference can be observed in the TMR values measured on the MTJ structure with the different thicknesses of the free layer. In particular, a TMR of 44% was measured at a free-layer thickness of 3 nm, while the TMR values were significantly lower at other thicknesses: 10% at 1.5 nm, 15% at 2 nm, and 32% at 3.5 nm. This difference underlines the importance of the thickness of the free ferromagnetic layer, and from our study, it is clear that 3 nm is the optimum thickness for both the free and reference layers, as it represents the maximum change in electrical resistance with respect to the applied magnetic field.

**Figure 10:**
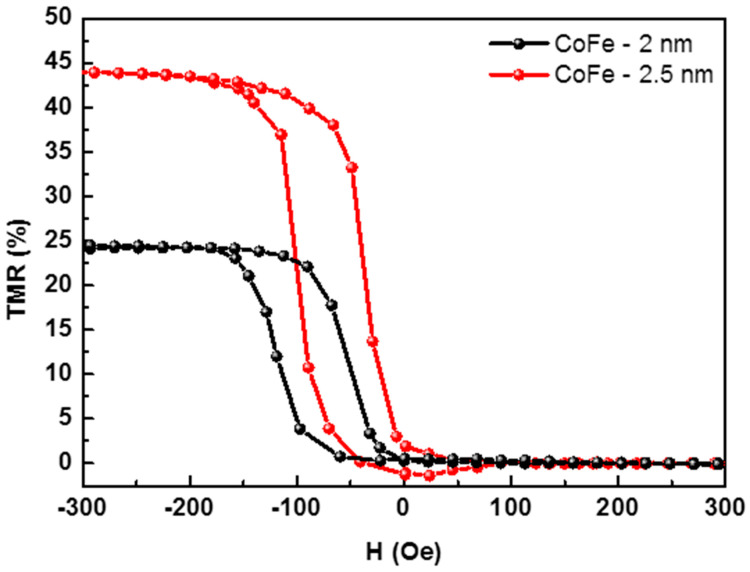
Magnetoresistance curves for MTJ structures as a function of CoFe layer thicknesses.

On the other hand, the influence of the pinned and seed CoFe layers on the magnetoresistance of the MTJ structure was also analyzed in this work. The orientation of the magnetic moments of these layers is oriented parallel to the applied magnetic field during deposition and significantly influences the variation in the electrical resistance. When comparing the variation in the electrical resistance with the applied magnetic field for CoFe film thicknesses of 2 and 2.5 nm (as shown in Figure 10), it was found that thinner CoFe films were associated with lower MR values. Therefore, 2.5 nm was determined to be the optimum thickness for the CoFe films.

Once the best conditions for the preparation of the thin films before the barrier layer have been determined, the deposition parameters and the thickness of the MgO layer were analyzed in order to obtain the most uniform, defect-free, and crystalline-oriented layer.

**Figure 11:**
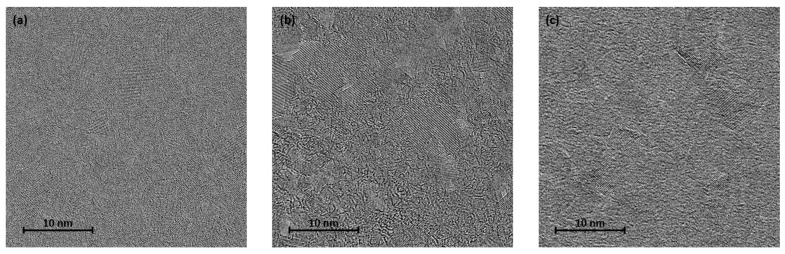
Transmission electron microscopy images of the MgO film deposited at rates: (**a**) 0.1 Å/s, (**b**) 0.2 Å/s, and (**c**) 0.3 Å/s.

The MgO layer was prepared by electron-beam evaporation, which is a reliable alternative to deposition of the barrier layer by RF sputtering or molecular-beam epitaxy (MBE) in MTJ-type magnetoresistive structures [30,31]. The quality of the MgO layer obtained by electron-beam evaporation strongly depends on the deposition rate. Analysis of the surface of the MgO film deposited at different rates (0.1 Å/s, 0.2 Å/s, and 0.3 Å/s) by transmission electron microscopy (TEM) revealed that the surface of the film is uniform at 0.1 Å/s (Figure 11a). As the rate increases, higher defect density and roughness are observed (Figure 11b,c); therefore, the deposition rate of the MgO film was fixed at 0.1 Å/s.

**Figure 12:**
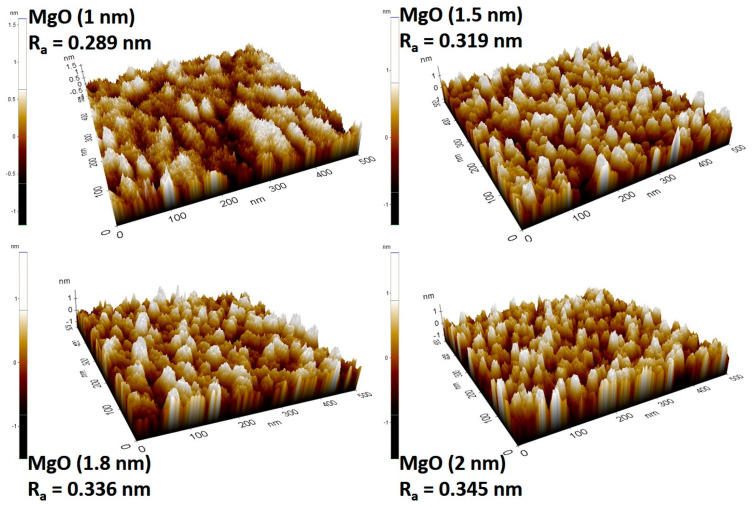
AFM topography images for different thicknesses of the MgO layer.

Of particular importance is the thickness of the MgO layer, which must be so thin that electrons can pass through the insulating layer due to the quantum tunnel effect. On the other hand, the thinner the MgO layer is, the more defects can occur in the crystal lattice due to the roughness of the previously deposited layers, and the electrons no longer tunnel coherently but rather in a different state, which leads to a reduction in the magnetoresistance of the MTJ structure. According to the literature [32,33], the MgO layer deposited on the amorphous CoFeB layer presents an amorphous structure when the thickness is less than 1 nm, while it presents a crystalline structure with preferential orientation (001) when the thickness is greater than 1 nm. Therefore, a series of four samples with different thicknesses of the MgO layer on the multilayer structure obtained under the previously determined conditions and thicknesses were subjected to AFM analysis (Figure 12).

As Figure 12 shows, an increase in roughness can be observed with the increasing thickness of the MgO layer. Considering that, on the one hand, the difference between the R_a_ value at thicknesses of 1 nm and 1.5 nm is very small and, on the other hand, the fact that 1 nm is the thickness at the limit between the amorphous form and the formation of the crystalline structure of the MgO layer [33], we consider 1.5 nm to be the optimal thickness of the barrier layer for achieving high performance of the MTJ structure.

### 3.4. Performance of the Magnetic-Tunnel-Junction-Based Structure

**Figure 13:**
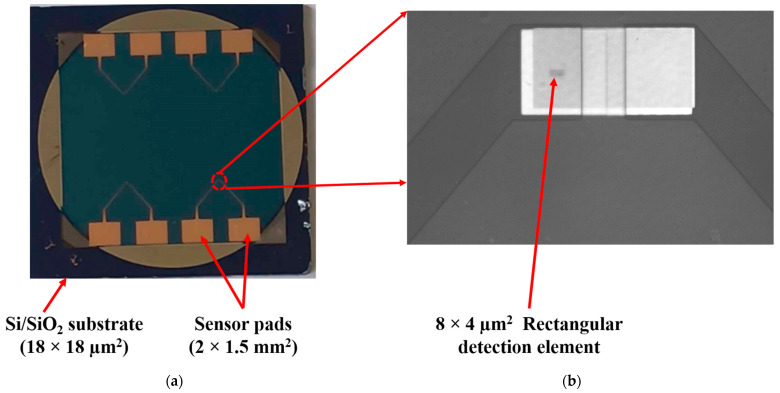
(**a**) Image of the chip with four sensors; (**b**) optical image of the 40 × 80 µm^2^ structure on which the rectangular pillar is defined.

To verify the effect of the optimized parameters used for obtaining the MTJ structure, we analyzed the sensor’s transfer curve before and after the optimization of the MTJ structure for several samples. The sensor transfer curve indicates how the resistance of the sensor changes with variations in the applied magnetic field, and the TMR reflects the sensitivity of the sensor to the magnetic field. For this purpose, the MTJ multilayer stacks were microfabricated in a CPP sensor configuration. For the measurements, the contacts are 2 × 1.5 mm^2^ squares, as illustrated in Figure 13a and the sensing element is a pillar with a rectangular surface of 4 × 8 µm^2^, as shown in the optical image of Figure 13b.

For the microfabrication process of the sensor, the following steps were carried out after the deposition of the MTJ structure:

1. Definition of an MTJ structure in a rectangular shape with dimensions of 40 × 80 μm^2^ using electron-beam lithography (EBL) and ion-beam etching (IBE). The detection element and the bottom contact are then defined on this rectangular structure.
2. Definition of the bottom contact by EBL and IBE followed by resist stripping.
3. Definition of the rectangular (4 × 8 μm^2^) detection element with EBL and IBE. In this stage, the surface of the bottom contact is protected by e-resist in the same lithography step as the detection element (pillar).
4. In order to prevent electrical contact between the bottom contact and the top surface of the MTJ, a 40 nm thick SiO_2_ insulating layer was deposited on the chip surface by sputtering, followed by a lift-off process.
5. Fabrication of the electrical contacts by laser-beam lithography and deposition of Ta (5 nm)/Cu (200 nm).

**Figure 14:**
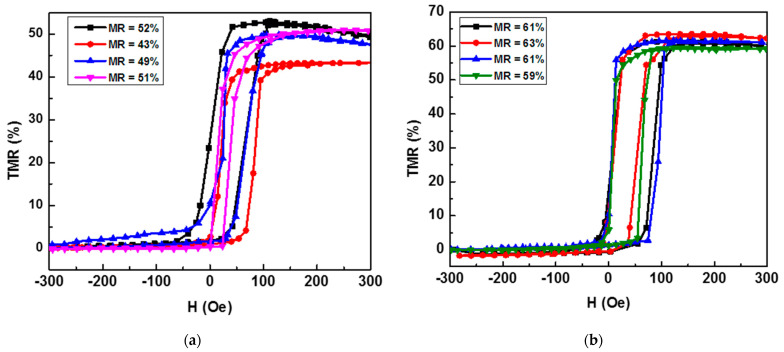
Sensor transfer curves before (**a**) and after (**b**) optimization of the component layer deposition parameters.

The fabricated sensors were magnetically annealed for one hour at 320 °C in a magnetic field of 6 kOe generated by a Helmholtz coil system connected to a bipolar source (Kepko BOP 100–10 MG) and measured with a Gaussmeter with Hall probe. The results presented in Figure 14 show a significant improvement in the tunnel magnetoresistance ratio, which increased on average by 10% when the optimized deposition parameters were used.

Although the obtained value is not very high, we believe that the small improvements in the method of producing the MTJ structure, as well as the adjustments in the layer thicknesses, have a positive effect on the performance of the resulting sensors based on the tunnel magnetoresistance effect. Based on these studies, we propose to continue with a more detailed analysis in the future to improve the sensitivity and reliability of MTJ magnetic sensors.

**Figure 15:**
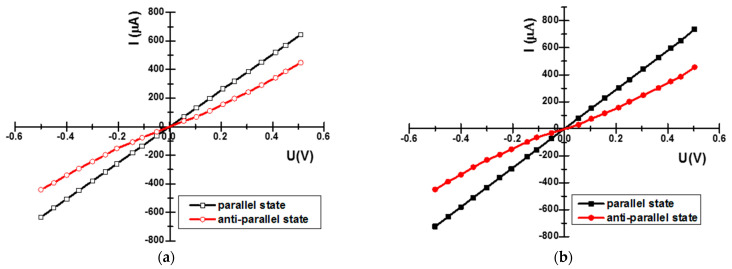
Current–voltage characteristics of the TMR-based sensor: (**a**) before optimization and (**b**) after optimization of the MTJ structure.

Because the optimization focused on not only increasing the TMR ratio but also decreasing the resistance-area (RA) product, in addition to analyzing the transfer curves, the current–voltage (I–V) characteristics of one MTJ structure before and the other after optimization were analyzed to obtain information about the efficiency of the optimization process and understand how it affects the overall functionality of the MTJ structure. The resistivity switching for the samples before and after optimization is shown in the current–voltage curves (I–V) in Figure 15.

The TMR ratio of the MTJ structures corresponds to the slopes of the I–V curves, which, in fact, indicate the actual tunnel magnetoresistance in the parallel and anti-parallel states. For the anti-parallel state, the curves clearly show the non-linear I–V behavior of the tunnel junction as being more evident for the optimized MTJ structure.

These results not only help improve our understanding of MTJ structures but also are promising for various applications. The adjustments of working gas pressure, discharge power, target–substrate distance, and film thickness jointly contributed to these advances.

## 4. Conclusions

By systematically optimizing the deposition parameters, we achieved about a 10% increase in the tunnel magnetoresistance of the MTJ structure. The precise control of the thin film properties achieved by adjusting the gas pressure, discharge power, and distance between target and substrate improves the uniformity of the film and reduces roughness, which is crucial for minimizing defects. Using magnetron sputtering and electron-beam evaporation, we have found the best conditions for depositing individual layers. After optimization, the structure retained its original configuration, (Ta (5 nm)/Ru (20 nm)/Ta (5 nm)/CoFe (2.5 nm)/IrMn (20 nm)/CoFe (2.5 nm)/Ru (0.85 nm)/CoFeB (3 nm)/MgO/CoFeB (3 nm)/Ta (5 nm)), with only the thickness of the MgO layer being reduced from 1.8 nm to 1.5 nm. The decrease in the working gas pressure from 5 mTorr to 3 mTorr after optimization indicates an improved efficiency. On the other hand, the discharge power in DC sputtering remained at 150 W, while in RF sputtering, it was adjusted from 120 W before optimization to 150 W after optimization. In addition, the distance between the target and the substrate was fixed at 20 cm throughout the optimization process.

Optimizing thin films thicknesses, particularly in the barrier layer (MgO) and synthetic antiferromagnetic structure, led to optimal tunneling behavior and antiferromagnetic coupling, enhancing spin-dependent transport properties. Despite these improvements, further investigation into factors like magnetic annealing and device geometry is needed, along with assessments of the scalability and reproducibility of the optimized parameters. In conclusion, systematic parameter optimization enhances MTJ device performance, offering the premise for more efficient magnetoresistive sensors.

## Figures and Tables

**Table 1:** Magnetic tunnel junction multilayers in order of their deposition from the Si/SiO_2_ wafer. The role and characteristics of each layer in the structure.

Thin Film	Role in MTJ Structure		Properties
**Ta**	Capping layer		▪ Protects against oxidation.
**CoFeB**	Free layer		▪ Amorphous after deposition; ▪ Crystallizes bcc (001) after thermal treatment.
**MgO**	Tunnel layer		▪ Crystalline FCC–orientation (001)
**CoFeB**	Synthetic antiferromagnetic (SAFM)	Reference layer	▪ Amorphous after deposition; ▪ Crystallizes bcc after thermal treatment.
**Ru**		Barrier layer in SAFM	▪ It ensures the antiparallel magnetization of the two ferromagnetic layers.
**CoFe**		Pinned layer	▪ Magnetically oriented during heat treatment in magnetic field.
**IrMn**	Antiferromagnetic layer		▪ Fixes the magnetization of the ferromagnetic layers and ensures the stability of the SAFM structure up to high magnetic fields; ▪ Thermally stable.
**CoFe**	Seed layer		▪ Magnetically oriented layer during heat treatment; ▪ Reduces mismatches between the crystalline networks of the buffer layer and the antiferromagnetic one; ▪ Ensures the type (111) texture of the IrMn substrate.
**Ta**	Buffer layer		▪ Reduces roughness.
**Ru**	Buffer layer		▪ Bottom contact
**Ta**	Buffer layer		▪ Ensures the adhesion of the structure; ▪ Small roughness.
**Si/SiO_2_**	Substrate		▪ Low roughness; ▪ Electrical insulator to prevent the structures from being short-circuited.

## Data Availability

The original contributions presented in the study are included in the article, further inquiries can be directed to the corresponding author.
